# A Herbal Medicine,* Gongjindan*, in Subjects with Chronic Dizziness (GOODNESS Study): Study Protocol for a Prospective, Multicenter, Randomized, Double-Blind, Placebo-Controlled, Parallel-Group, Clinical Trial for Effectiveness, Safety, and Cost-Effectiveness

**DOI:** 10.1155/2017/4363716

**Published:** 2017-12-13

**Authors:** Seungwon Shin, Jinyoung Kim, Ami Yu, Hyung-Sik Seo, Mi-Ran Shin, Jae-Heung Cho, Gilhee Yi, Seung-Ug Hong, Euiju Lee

**Affiliations:** ^1^Department of Clinical Korean Medicine, Graduate School, Kyung Hee University, 26 Kyungheedae-ro, Dongdaemun-gu, Seoul 02447, Republic of Korea; ^2^Korean Medicine Clinical Trial Center, Kyung Hee University Korean Medicine Hospital, 23 Kyungheedae-ro, Dongdaemun-gu, Seoul 02447, Republic of Korea; ^3^Department of Korean Medical Ophthalmology & Otolaryngology & Dermatology, School of Korean Medicine, Pusan National University, 20 Geumo-ro, Mulgeum-eup, Yangsan, Gyeongsangnam-do 50612, Republic of Korea; ^4^Department of Sasang Constitutional Medicine, College of Oriental Medicine, Semyung University, 65 Semyung-ro, Jecheon, Chungcheongbuk-do 27136, Republic of Korea; ^5^Department of Korean Rehabilitation Medicine, College of Korean Medicine, Kyung Hee University, 23 Kyungheedae-ro, Dongdaemun-gu, Seoul 02447, Republic of Korea; ^6^Department of Oriental Medicine Ophthalmology & Otolaryngology & Dermatology, College of Oriental Medicine, Dongguk University, 27 Dongguk-ro, Ilsandong-gu, Goyang, Gyeonggi-do 10326, Republic of Korea; ^7^Department of Sasang Constitutional Medicine, College of Korean Medicine, Kyung Hee University, 23 Kyungheedae-ro, Dongdaemun-gu, Seoul 02447, Republic of Korea

## Abstract

This study protocol aims to explore the effectiveness, safety, and cost-effectiveness of a herbal medication,* Gongjindan* (GJD), in patients with chronic dizziness. This will be a prospective, multicenter, randomized, double-blind, placebo-controlled, parallel-group, clinical trial. Seventy-eight patients diagnosed with Meniere's disease, psychogenic dizziness, or dizziness of unknown cause will be randomized and allocated to either a GJD or a placebo group in a 1 : 1 ratio. Participants will be orally given 3.75 g GJD or placebo in pill form once a day for 56 days. The primary outcome measure will be the Dizziness Handicap Inventory score. Secondary outcome measures will be as follows: severity (mean vertigo scale and visual analogue scale) and frequency of dizziness, balance function (Berg Balance Scale), fatigue (Fatigue Severity Scale) and deficiency pattern/syndrome (qi blood yin yang-deficiency questionnaire) levels, and depression (Korean version of Beck's Depression Inventory) and anxiety (State-Trait Anxiety Inventory) levels. To assess safety, adverse events, including laboratory test results, will be monitored. Further, the incremental cost-effectiveness ratio will be calculated based on quality-adjusted life years (from the EuroQoL five dimensions' questionnaire) and medical expenses. Data will be statistically analyzed at a significance level of 0.05 (two-sided). This trial is registered with ClinicalTrials.gov NCT03219515, in July 2017.

## 1. Introduction

Dizziness is a typical manifestation of various neurological diseases. It is mostly related to vestibular disorders of the inner ear, such as benign paroxysmal positional vertigo (BPPV), vestibular neuritis, or Meniere's disease (MD) [1]. However, the symptom may also arise from some conditions involving the central nervous system (e.g., cerebral stroke, vertebrobasilar insufficiency), cardiovascular diseases (e.g., arrhythmia, orthostatic hypotension) [2], or even certain psychiatric conditions [3]. In some cases, chronic and recurrent dizziness fails to receive a diagnosis and remains as unspecified or undefined dizziness in clinical records.

According to studies investigating the etiology of recurrent dizziness, 12.5% of cases were attributed to MD [4], 15% to psychogenic dizziness [5], and 10–52% to dizziness of unknown cause [5, 6]. Public healthcare data systems also show the incidence of dizziness has increased by approximately 20% in the past 5 years [7].

Otorhinolaryngologists prescribe vasodilators or diuretics to patients with MD. For example, betahistine is frequently used as a full agonist of the H1 receptors located in blood vessels in the inner ear, which can increase local vasodilation and help alleviate symptoms [8]. Moreover, diuretics are sometimes used to treat endolymphatic hydrops. However, there is no confirmatory evidence of the utility of these treatments [4]. For psychogenic dizziness, psychiatrists administer to patients antianxiety agents or antidepressants, accompanied by cognitive-behavioral therapy [3], which also lack clinical verification. If the dizziness is due to unknown causes, drugs, such as meclizine, clonazepam, or lorazepam, or rehabilitation therapy is prescribed, which mostly rely on anecdotal evidence [6].

Due to these limitations, many patients with dizziness visit practitioners of traditional medicine in Korea. Traditional Korean Medicine (TKM) doctors prescribe various herbal medications to these patients, based on syndrome/pattern identification [9].* Gongjindan* (GJD) is one of the most preferred options. A classic Korean book of traditional medicine,* Dongeuibogam*, suggests that GJD can be prescribed for liver-deficiency syndrome/pattern with symptoms of dizziness/vertigo, dim eyes, a pale face, and/or weak muscles [10, 11]. However, the modern scientific level of the evidence for GJD remains low.

Most studies have been* in vivo* or* in vitro* experiments that have demonstrated the efficacy of GJD in ameliorating memory impairments [12, 13] or fatigue [14], its antioxidant effects [15], neuroregulatory effects for transient middle cerebral artery occlusion [16] or Alzheimer's disease [17], and lipid-lowering effect in hyperlipidemia [18]. Clinical studies have been rarer. A retrospective study for Alzheimer's disease [19] and a case report for dizziness of patients with anemia [20] have been published. Only one randomized controlled trial (RCT) with GJD has so far been planned with fatigue patients [21], which has not announced its results yet. Therefore, the effectiveness of GJD should be explored clinically with qualified RCTs using a placebo for comparison. This study would be the first RCT for chronic dizziness to explore the clinical effectiveness, safety, and cost-effectiveness of a herbal drug, GJD pills.

This study protocol is primarily aimed at exploring the effectiveness of GJD in patients with chronic dizziness, compared to placebo. Additionally, the safety and cost-effectiveness of GJD will be investigated.

## 2. Materials and Methods

This study protocol has been designed to conform with Standard Protocol Items: Recommendations for Interventional Trials (SPIRIT), 2013 statement [22], and the elaborated Consolidated Standards of Reporting Trials (CONSORT) statement for herbal interventions [23].

### 2.1. Objectives and Hypothesis

The primary objective of this study is to evaluate the effect of GJD administration for 8 weeks, compared to placebo, on chronic dizziness (MD, psychogenic dizziness, or dizziness of unknown cause). The alternative hypothesis is that the mean difference between Dizziness Handicap Inventory (DHI) scores at baseline and endpoint in the GJD group will not be equal to that in the placebo group.

Additionally, the changes in dizziness severity and frequency will be evaluated with validated scales. Further, the levels of fatigue, depression, anxiety, and quality of life (QoL) will be compared between the two groups posttreatment. The safety of GJD administration will be examined based on laboratory tests and adverse events (AEs). Finally, the cost-effectiveness of GJD will be evaluated with quality-adjusted life years (QALY).

### 2.2. Study Design

**Figure 1:**
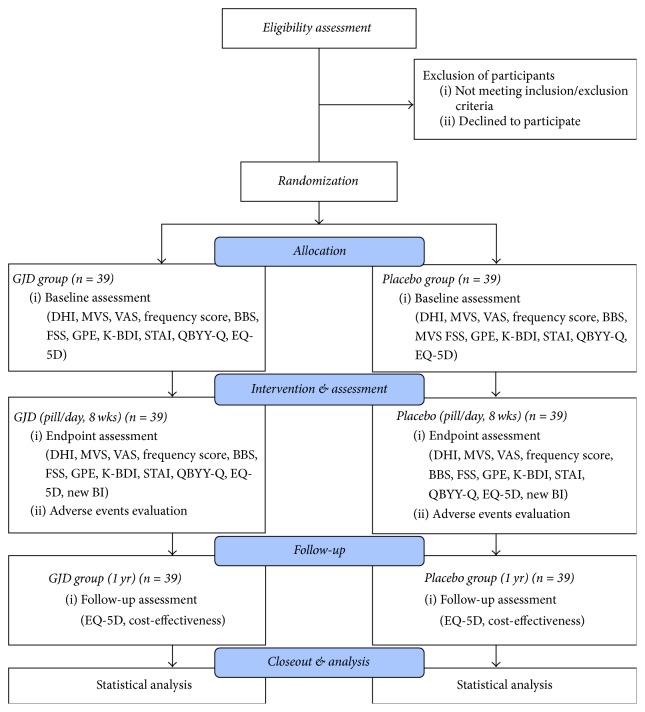
Study flowchart. BBS, Berg Balance Scale; BI, Blinding Index; CNS, central nervous system; DHI, Dizziness Handicap Inventory; EQ-5D, EuroQoL five dimensions' questionnaire; FSS, Fatigue Severity Scale; GJD,* Gongjindan*; GPE, Global perceived effect; K-BDI, Korean version of Beck's Depression Inventory; MVS, mean vertigo scale; QBYY-Q, qi blood yin yang-deficiency questionnaire; STAI, State-Trait Anxiety Inventory; VAS, visual analogue scale; wks, weeks; yr, year.

This study will be a prospective, multicenter, randomized, double-blind, placebo-controlled, parallel-group, clinical trial. Seventy-eight patients diagnosed with MD, psychogenic dizziness, or dizziness of unknown cause, with a DHI score ≥ 24 at baseline, and having complained of dizziness for more than 1 month will be randomized and allocated to either the GJD or placebo group in a 1 : 1 ratio. They will be orally given 3.75 g GJD or placebo in pill form once a day for 8 weeks (56 pills in total). To collect data for the cost-effectiveness analysis, we will follow up the patients for up to 12 months from randomization. The study flowchart is displayed in diagram form in Figure 1 and the specified timetable is presented in Table 1.

### 2.3. Setting and Recruitment

Four TKM hospitals in the Republic of Korea, that is, Kyung Hee University Korean Medicine Hospital in Seoul, Dongguk University Ilsan Oriental Hospital in Goyang (Gyeonggi-do), Pusan National University Korean Medicine Hospital in Yangsan (Gyeongsangnam-do), and Semyung University Korean Medicine Hospital in Chungju (Chungcheongbuk-do), will enroll eligible participants. Participants will be recruited mostly from the respective outpatient clinics. Moreover, study advertisements will be posted on webpages and notice boards.

### 2.4. Ethical Review and Trial Registration

This protocol (version 2.5) and the informed consent forms have been peer-reviewed and approved by the Institutional Review Board of Kyung Hee University Korean Medicine Hospital (approval number KOMCIRB-170417-HRBR-012) following the scientific content and ethical compliance with regulations, that is, Good Clinical Practice and relevant laws by Ministry of Food and Drug Safety in Korea. The study has been registered at ClinicalTrials.gov in July 2017 (identifier: NCT03219515).

### 2.5. Eligibility Criteria

Participants will be included if they satisfy all of the following criteria:

1. Age between 20 and 79 years, of either sex
2. Dizziness originating from MD, psychogenic cause, or unknown cause
3. Recurring symptom of dizziness for more than 1 month
4. DHI score ≥ 24 at baseline
5. Liver-deficiency pattern/syndrome identified by TKM doctors
6. Willingness to provide written informed consent.

The targeted scope of chronic dizziness has been established based on a preceding acupuncture trial [24]. The liver-deficiency syndrome is defined as having all of the main symptoms and at least two of the secondary symptoms [10, 25]. The main symptoms include dizziness, dim eyes, and pale face. The secondary symptoms include lack or a momentary loss of vision, numbness at extremities, muscular spasm, twitching and/or cramping, hypochondriac pain, dry or pale fingernails, toenails, or lips, tinnitus, and scant menstruation. Two TKM doctors who have 1 year or more of clinical experience will independently identify the pattern/syndrome, and only the patients diagnosed with consistent liver-deficiency pattern/syndrome will be enrolled in the study.

Participants will be excluded if any of the following criteria is applicable:

1. Dizziness attributable to vestibular disorders (e.g., benign paroxysmal positional vertigo, peripheral vestibulopathy, labyrinthitis, and vestibular neuronitis)
2. Dizziness attributable to central nervous system (CNS) disorders (e.g., cerebellar ataxia, stroke, demyelination, vertebrobasilar insufficiency, seizure, increased intracranial pressure, Parkinson's disease, and migraines)
3. Cervicogenic dizziness
4. Dizziness attributable to cardiovascular disorders (e.g., arrhythmia, heart valvular disease, anemia, orthostatic hypotension, and coronary artery disease)
5. Any active or uncontrolled disease that might cause dizziness (e.g., uncontrolled diabetes mellitus, hypertension, and respiratory or endocrinological disorders)
6. Dizziness attributable to medication side effects
7. Severe chronic or terminal diseases (malignant cancer, tuberculosis, etc.)
8. Intake of antivertiginous drugs that cannot be discontinued
9. Following physiotherapy, manual therapy (e.g., vestibular rehabilitation), and/or cognitive-behavioral therapy for the treatment of dizziness
10. Aspartate aminotransferase (AST), alanine aminotransferase (ALT), blood urea nitrogen (BUN), or creatinine > 3 × upper limit of normal range at baseline
11. Women of (suspected) pregnancy or breast-feeding
12. Allergic reactions to the study medications
13. Suspicion of alcohol and/or drug abuse
14. Enrollment in another clinical study presently or within 30 days prior to the initial administration of the study medications
15. Difficulty in reliably communicating with the investigators or likelihood of inability to follow instructions
16. Reasons for ineligibility of participation judged by investigators.

### 2.6. Dropout Criteria

Already enrolled participants will be dropped out if they withdraw their consent for participation, are lost to follow-up, cannot continue participation due to adverse events or complications, consume disallowed medications, or markedly deviate from the study protocol.

### 2.7. Randomization, Allocation, and Blinding

An independent statistician (A. Yu) will generate random sequence numbers via SAS® 9.4 software (SAS Institute Inc., Cary, NC). Stratified randomization will be performed in blocks in order to keep the sizes of both arms similar with the strata of the study sites. The random number table will be delivered from the statistician to a pharmaceutical company, where GJD and placebo pills will be produced.

The investigational products labeled with random codes will be provided to a pharmacist, who will dispense them to the enrolled participants. According to the order of enrollment, participants will be allocated to either the GJD group or the placebo group in a 1 : 1 ratio. As only the statistician and the pharmaceutical company will have access to the random table throughout the study, allocation will stay concealed.

Since we are going to use placebo pills that look, taste, and smell similar to GJD pills, both investigators and patients will stay blinded until the completion of the study. Only clinical emergency due to serious AEs will break the code and blinding.

### 2.8. *Gongjindan* (GJD)

The GJD group will be orally given 3.75 g of GJD in pill form (product name: Iksu Gongjindan), on an empty stomach every morning for 8 weeks (56 days). This has been determined based on clinical experience within the dose regimen (1 to 3 oral administrations per day) of GJD pills approved by the Ministry of Food and Drug Safety in Korea. The manufacturer, Iksu Pharmaceutical Co. Ltd. (Gwangju, Republic of Korea). obtained Good Manufacturing Practice authorization from the Ministry of Food and Drug Safety in Korea. The GJD pill and its ingredients have also been registered with the same governmental authority.

GJD is composed of six herbs (with country of origin and material standard; KHP, Korean Herbal Pharmacopoeia; KP, Korean Pharmacopoeia): Cervi Parvum (*Cervus elaphus* Linné, family Cervidae, Russia, KHP) 444.33 mg, Angelica Gigas Root (*Angelica gigas* Nakai, family Umbelliferae, Korea, KP) 444.33 mg, Cornus Fruit (*Cornus officinalis* Siebold et Zuccarini, family Cornaceae, China, KP) 444.33 mg, Ginseng (*Panax ginseng* C. A. Meyer, family Araliaceae, Korea, KP) 444.33 mg, Steamed Rehmannia Root (*Rehmannia glutinosa* Liboschitz ex Steudel, family Scrophulariaceae, China, KP) 444.33 mg, and Musk (*Moschus moschiferus* Linné, family Moschidae, Russia, KHP) 74 mg.

After the herbs are weighed, they are pulverized, sterilized, and mixed with the excipient (corn starch 233.33 mg), binders (glycerin 550 mg and suitable amount of honey), and preservative (sodium benzoate 2.25 mg). The mixture is formed into a pill (3.75 g/pill) and covered with gilt paper. Separately packaged and labeled GJD will be directly delivered to the pharmacists of each study site from the pharmaceutical company. A voucher specimen will be retained and kept with the manufacturer.

### 2.9. Placebo

Since there have been no clinical trials to show the effectiveness and/or safety of GJD pills over placebo, we have decided to adopt placebo control for this dizziness trial. Placebo pills will be made by the same pharmaceutical company as the GJD pills. They will be made similar in appearance, taste, and odor to the GJD pills, containing excipients (corn starch 997.76 mg, sodium carboxymethyl cellulose 80 mg, lactose hydrate 1.402 mg, and citric acid hydrate 40 mg), coloring agents (sepia color 10 mg and caramel color 300 mg), binders (glycerin 300 mg and honey 400 mg), flavoring agents, and preservative (sodium benzoate 2.24 mg) with gilt-paper covering. The entire manufacturing process will follow the relevant guidelines of the Korean Ministry of Food and Drug Safety.

The placebo group will be orally given 3.75 g of placebo pill on an empty stomach every morning for 8 weeks (56 days).

### 2.10. Concomitant Medications

All of the concomitant medications will be recorded throughout the trial. No other drugs (betahistine, difenidol, flunarizine, cinnarizine, dimenhydrinate, gingko, meclizine, metoclopramide, scopolamine, pentoxifylline, perphenazine, ergoloid mesylate, droperidol, phenobarbital, prochlorperazine, promethazine, trimethobenzamide, or vertigoheel, etc.) or herbal drugs to manage the symptom of dizziness will not be allowed. Moreover, other therapies for dizziness, like physical therapy, rehabilitation, acupuncture, electroacupuncture, pharmacoacupuncture, moxibustion, cupping, and cognitive-behavioral therapies, will be disallowed during the study.

The following drugs, which can induce drug-related dizziness, will be closely monitored: *α*1-adrenergic antagonists, alcohol, aminoglycosides, anticonvulsants, antidepressants, anti-Parkinson medication, antipsychotics, *β*-blockers, calcium channel blockers, class 1a antiarrhythmics, digitalis glycosides, diuretics, narcotics, oral sulfonylurea, vasodilators, anticoagulants, antidementia agents, antihistamines (sedating), antirheumatic agents, anti-infectives (anti-influenza agents, oral antifungals, quinolones), antithyroid agents, anxiolytics, attention-deficit/hyperactivity disorder agents, cholesterol-lowering agents, bronchodilators, skeletal muscle relaxants, urinary and gastrointestinal antispasmodics, and so on.

### 2.11. Screening Process

After a TKM doctor obtains informed consent from a patient, he/she will go through an extensive screening process for the eligibility decision. Height, weight, body mass index, vital signs (blood pressure, pulse, and body temperature), and electrocardiogram will be measured. Furthermore, blood and urine laboratory tests will be performed, including pregnancy testing. History of medical diseases and drugs will be recorded. If the patient was under administration of any of the drugs disallowed in this study, he/she will be able to participate after a 2-week wash-out period. To rule out untargeted diseases related to chronic dizziness, as listed in the exclusion criteria, we are going to test for CNS abnormalities (deep tendon reflex, Hoffman sign, Babinski sign, ankle clonus, finger to nose, finger to finger, rapid alternating movement, heel to shin, Romberg's test, Brudzinski sign, Kernig sign, Naffziger test, etc.). Additionally, spontaneous, gaze-evoked, positional, and positioning (Dix-Hallpike) nystagmus will be tested for, too. Finally, two TKM doctors will judge if the patient has liver-deficiency pattern/syndrome or not, and those that will receive a consistent diagnosis will be considered to be eligible. The whole process is depicted in Table 1.

### 2.12. Outcomes

#### 2.12.1. Primary Outcome

*Dizziness Handicap Inventory (DHI)*. DHI was developed to evaluate functional, emotional, and physical impairments due to dizziness [26]. This widely used scale for patients with dizziness is a patient-rated outcome (PRO) and has been validated in Korean [27]. The scale consists of 25 items (0, 2, or 4 scores/item, 0–100 scores). The primary endpoint is the mean difference of DHI total score between the baseline and the endpoint of the treatment period. Secondarily, the before-and-after effect of GJD between the baseline and every visit will also be investigated.

#### 2.12.2. Secondary Outcomes

The following outcomes will be evaluated at baseline, mid-term point, and the endpoint of the treatment. The mean differences of each scale score from the baseline and each measurement point will be compared between the GJD and the placebo groups.

*Mean Vertigo Score (MVS)*. MVS is a PRO assessing the intensity of dizziness and has been employed in various clinical trials [28–35]. It includes six items for symptom types and six items for symptom provoking conditions. Patients answer on a scale from 0 (none) to 4 (very strong) and the final score is calculated as the sum divided by 12 (0–4 scores).

*Visual Analogue Scale (VAS)*. The VAS was used in the previous studies on dizziness [36–41]. This scale consists of a 10 cm line, where 0 represents “not dizzy at all” and 10 “dizziest I can imagine being.”

*Dizziness Frequency*. Patients will be asked how many episodes of dizziness they experience. The frequency will be scored as 0 (none), 1 (less than once a month), 2 (1–4 episodes/month), 3 (1–4 episodes/week), 4 (once a day), or 5 (more than twice a day), as per the notation used in the previous studies [38–41].

*Berg Balance Scale (BBS)*. The BBS was originally developed for elderly patients with balance impairment; however, it has been used to assess overall balance ability regardless of age. Its reliability [42, 43], validity [44], and sensitivity [45] have been verified and it has been validated in Korean [46]. Patients will be asked to perform 14 tasks and each task will be evaluated separately.

*Fatigue Severity Scale (FSS)*. GJD is widely administered to patients with chronic fatigue. In this study, we are going to assess the patients' fatigue level with the FSS. This 7-point Likert scale (nine items, 9–63 score range) is a PRO and it has been validated [47].

*Global Perceived Effect (GPE)*. GPE is a validated scale evaluating a patient's perception of symptom worsening or improvement around a specific timepoint [48]. This scale has also been used in clinical studies on dizziness [38, 40, 41], and it consists of seven options (1 for completely recovered to 7 for worst ever).

Based on the previous study showing that the patients with dizziness can become markedly depressed and anxious [49], the following scales will also be included.

*Korean Version of Beck's Depression Inventory (K-BDI)*. The K-BDI is a PRO evaluating the severity of depression with 21 items (0 to 3/points, 63 points in total). The Korean version of this scale has been validated [50, 51].

*State-Trait Anxiety Inventory (STAI)*. The STAI assesses the level of anxiety in the subdomains of state and trait. Each subdomain includes 20 items and each item has the options of 1–4 points (20–80 scores/subdomain). Its reliability and validity have been shown [52].

*Qi Blood Yin Yang-Deficiency Questionnaire (QBYY-Q)*. GJD is a herbal medication prescribed to patients with liver-deficiency pattern/syndrome in TKM. Therefore, we assumed that the level of deficiency pattern/syndrome could vary between groups at the end of the intervention. QBYY-Q is a PRO to evaluate the severity of qi-, blood-, yin-, and yang-deficiency pattern/syndrome [53, 54]. A previous clinical study on chronic fatigue demonstrated the reliability and validity of this scale [55]. The scale consists of 32 items and each item is rated from 0 to 3 points.

#### 2.12.3. Blinding Assessment

This is a double-blind clinical trial with placebo control. Both study participants and TKM doctors will remain blind until the end of the trial. At the end of treatment of each participant, they will be asked in which group they think they belonged to, the GJD group, placebo group, or unknown, and the new Blinding Index (BI) will be calculated following [56]. The index score varies within 1 (complete lack of blinding), 0 (consistent with perfect blinding), or −1 (guessed they were in the opposite group).

#### 2.12.4. Safety Assessment

The AEs will be monitored during the whole treatment period. The following adverse reactions are expected: anorexia, discomfort feeling of stomach area, nausea, vomiting, diarrhea, or rash on skins. The intensity and frequency of each episode will be recorded. Moreover, laboratory tests will be performed at baseline and the endpoint. Complete blood cell tests (red blood cell, hemoglobin, hematocrit, platelet, and white blood cell), serum biochemical tests (creatinine, BUN, ALT, AST, and glucose), and urinalysis (pH, specific gravity, glucose, ketone, red blood cell, nitrate, total protein, bilirubin, urobilinogen, and white blood cell) will be performed. If serious AEs occur, postmanagement will be properly conducted.

#### 2.12.5. Cost-Effectiveness Assessment

The EuroQoL five dimensions' questionnaire (EQ-5D), consisting of two parts, that is, EQ-5D-5L and EQ VAS, will be used to assess quality of life, based on which QALY will be calculated. The Korean version of EQ-5D scale has been studied on its validity and reliability [57]. Also, direct costs (medical and nonmedical expenses) incurred in treating dizziness during the treatment (8 weeks) and follow-up (up to 12 months from randomization) periods will be investigated (Table 1). Medical fee signifies the cost paid in hospitals or clinics. Nonmedical fee signifies transportation and time costs that patients would incur for dizziness treatment. Based on the calculated QALY and fees, the incremental cost-effectiveness ratio (ICER) will be estimated (ICER = Δ  cost/Δ effectiveness). The ICER signifies the amount of cost necessary to improve by 1 unit of effectiveness.

### 2.13. Sample Size Calculation

To calculate the effect size, we used a preceding RCT on acupuncture therapy for MD with DHI assessment, as there have been no clinical studies for GJD effectiveness in chronic dizziness [58]. The null hypothesis is that *μ*_GJD_ = *μ*_placebo_, while the alternative hypothesis is *μ*_GJD_ ≠ *μ*_placebo_, where *μ* denotes the mean difference of DHI score between the baseline and endpoint. With the mean difference (−3.63) and the standard deviation (4.84), under the assumption of correlation coefficient (0.1), the sample size was calculated as 29 per group (two-sided, *α* = 0.05 and 1 − *β* = 0.8). Therefore, we are going to recruit 78 participants for both groups (39 per group) under a 25% anticipated dropout rate.

### 2.14. Statistical Analysis

The primary and secondary variables for the effect assessments will be statistically analyzed by analysis of covariance (ANCOVA) or rank ANCOVA with group and site as covariates. If any demographic characteristics show significant difference between groups, those variables will also be used as covariates. When there is a significant interaction between diagnosis (MD, psychogenic dizziness, or dizziness of unknown cause) and group, subgroup analysis will be performed.

The confidence interval (CI) of BI will be addressed and blinding will be deemed successful when the CI includes the zero value. For safety assessment, the chi-squared test or Fisher's exact test will be performed with the proportions of AEs, while lab results will be compared between groups by chi-squared test (proportions of abnormal results) or McNemar's test (before-and-after comparison within a group). An independent statistician (A. Yu) will analyze any data with a significance level of 0.05 (two-sided) with SAS 9.4 software (SAS Institute Inc.).

The primary analysis will be performed with the full analysis set, which is defined as the group of participants who were prescribed at least once or more the GJD or placebo pills and for whom DHI was evaluated more than once. The missing data will be imputed with the last observation carried forward method. The per protocol set will be additionally analyzed, which includes only the participants who complete all the scheduled visits. We are going to include any participants who took at least one pill of the study drugs for the safety assessment.

## 3. Results and Discussion

This study is a prospective, multicenter, randomized, double-blind, placebo-controlled, parallel-group, clinical trial to explore the effectiveness, safety, and cost-effectiveness of a herbal drug, GJD, for patients with chronic dizziness attributable to MD, psychogenic dizziness, or dizziness of unknown cause. We are going to administer to patients GJD or placebo pills for 8 weeks and follow them up for 12 months. Four TKM hospitals in Korea will enroll 78 participants.

Dizziness may ensue from a variety of central or peripheral diseases and in some cases as a drug complication. This may complicate the diagnostic process. This study will include patients diagnosed with MD, psychogenic dizziness, or dizziness of unknown cause, which may create an issue with homogeneity. However, there were previous clinical studies which also focused on the symptom of dizziness with varied origin to demonstrate new efficacy [28, 35], including an acupuncture study for chronic dizziness [24], which has meaningfully been referred for this trial.

GJD is a widely prescribed herbal pill in Korea, but it is mostly used for patients with severe deficiency pattern/syndrome, especially those complaining of marked fatigue [14, 21]. However, based on the seminal work* Dongeuibogam* [10], we expect GJD to also manage chronic dizziness.

This RCT included various diseases (MD, psychogenic dizziness, or dizziness of unknown cause) inducing dizziness, which might reduce the level of homogeneity of patient population. The investigators discussed this issue, which might affect significantly the study results in the future. However, it was taken into consideration that this trial should reflex the real clinical practices as much as possible, which means that GJD is a herbal drug and it has been described based on pattern/syndrome identification in TKM. So, we think the screening process is one of the key parts for this trial to include eligible participants. Investigators and coordinators should be trained with written standard operating procedures for the trial before enrolling the first participants, since this study is a multicenter trial, to control the high quality throughout the whole process. Also, an independent monitor will regularly visit all the hospitals for auditing ethical and scientific issues.

## 4. Conclusions

We are going to conduct a prospective, multicenter, randomized, double-blind, placebo-controlled, parallel-group, clinical trial on patients with chronic dizziness to evaluate the effectiveness, safety, and cost-effectiveness of a herbal drug, GJD. This study has been approved by an ethics committee and registered in the WHO international clinical trials registry platform (CRIS), in the Republic of Korea.

## Figures and Tables

**Table 1:** Study timetable.

	Screening	Randomization	Treatment				Follow-up		
Visits	V1	V2	*V3*	*V4*	*V5*	*V6*	*V7*	*V8*	*V9*
Timepoint	*Within 14 D*	*Day 0*	*14 D ± 3 D*	*28 D ± 3 D*	*42 D ± 3 D*	*56 D ± 3 D*	*4 M ± 1 W*	*8 M ± 1 W*	*12 M ± 1 W*
Enrollment:									
*Informed consent*	×								
*Demographics*	×								
*Vital sign*	×	×	×	×	×	×			
*Electrocardiography*	×					×			
*Lab test*	×					×			
*Medical history*	×								
*Concomitant medication*	×	×	×	×	×	×			
*Pregnancy test*	×								
*CNS examination*	×								
*Nystagmus examination*	×								
*Eligibility assessment*	×								
*Random allocation*		×							
Interventions:									
*GJD (1 pill/day)*			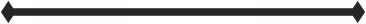						
*Placebo (1 pill/day)*			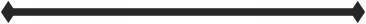						
Assessments:									
*DHI*	×	×	×	×	×	×			
*MVS*		×		×		×			
*VAS*		×		×		×			
*Frequency*		×		×		×			
*BBS*		×		×		×			
*FSS*		×		×		×			
*GPE*						×			
*K-BDI*		×		×		×			
*STAI*		×		×		×			
*QBYY-Q*		×		×		×			
*EQ-5D*		×		×		×	×	×	×
*Cost information*			×	×	×	×	×	×	×
*New BI*						×			
*Adverse events*			×	×	×	×			

BBS, Berg Balance Scale; BI, Blinding Index; D, day(s); DHI, Dizziness Handicap Inventory; EQ-5D, EuroQoL five dimensions' questionnaire; FSS, Fatigue Severity Scale; GJD, *Gongjindan*; GPE, global perceived effect; K-BDI, Korean version of Beck's Depression Inventory; M, month(s); MVS, mean vertigo scale; QBYY-Q, qi blood yin yang-deficiency questionnaire; STAI, State-Trait Anxiety Inventory; VAS, visual analogue scale; W, week(s).

## Abbreviations

AST: Aspartate aminotransferase
AE: Adverse event
ALT: Alanine aminotransferase
ANCOVA: Analysis of covariance
BBS: Berg Balance Scale
BI: Blinding Index
BPPV: Benign paroxysmal positional vertigo
BUN: Blood urea nitrogen
CI: Confidence interval
CONSORT: Consolidated Standards of Reporting Trials
CRIS: Clinical Research Information Service
DHI: Dizziness Handicap Inventory
e: Central nervous system
EQ-5D: EuroQoL five dimensions' questionnaire
FSS: Fatigue Severity Scale
GJD: * Gongjindan*
GPE: Global perceived effect
K-BDI: Korean version of the Beck's Depression Inventory
KHP: Korean Herbal Pharmacopoeia
KP: Korean Pharmacopoeia
MD: Meniere's disease
MVS: Mean vertigo score
PRO: Patient-rated outcome
QALY: Quality-adjusted life year
QBYY-Q: Qi blood yin yang-deficiency questionnaire
QoL: Quality of life
RCT: Randomized controlled trial
SPIRIT: Standard Protocol Items: Recommendations for Interventional Trials
STAI: State-Trait Anxiety Inventory
TKM: Traditional Korean Medicine
VAS: Visual analogue scale.

## References

[1] Kerber K. A., Baloh R. W. (2011). The evaluation of a patient with dizziness. *Neurology: Clinical Practice*.

[2] Lee D. K. (2014). Clinical understanding to dizziness in the elderly. *Korean Journal of Clinical Geriatrics*.

[3] Lee K. K. (2016). Psychogenic dizziness for psychiatrists in Korea. *Korean Journal of Psychosomatic Medicine*.

[4] Kwon S. Y., Hong S. K. (2012). Evaluation and treatment of Meniere’s disease. *Research in Vestibular Science*.

[5] Kroenke K., Lucas C. A., Rosenberg M. L. (1992). Causes of persistent dizziness: A prospective study of 100 patients in ambulatory care. *Annals of Internal Medicine*.

[6] Oh S. Y. (2010). Diagnosis and treatment of chronic dizziness. *Research in Vestibular Science*.

[7] (2017). *Review Assessment Service, Healthcare Bigdata Hub, Wonju: Health Insurance Review Assessment Service (Republic of Korea*.

[8] Sokolova L., Hoerr R., Mishchenko T. (2014). Treatment of vertigo: a randomized, double-blind trial comparing efficacy and safety of ginkgo biloba extract EGb 761 and betahistine. *International Journal of Otolaryngology*.

[9] Oh J. M., Eom T. M., Choi K. E. (2015). Study of the patients with dizziness who visited the Korean medicine hospital. *Korean Journal of Oriental Physiology Pathology*.

[10] Heo J. (2014). *Dongeuibogam*.

[11] Lee J. H., Jo D. C., Kim C. G. (2013). A literature review of effectiveness on the Gongjin-dan (Gongchen-dan). *Journal of Korean Medicine Rehabilitation*.

[12] Moon E., Her Y., Lee J. B. (2009). The multi-herbal medicine Gongjin-dan enhances memory and learning tasks via NGF regulation. *Neuroscience Letters*.

[13] Lee J.-S., Hong S.-S., Kim H.-G. (2016). Gongjin-Dan enhances hippocampal memory in a mouse model of scopolamine-induced amnesia. *PLoS ONE*.

[14] Hong S.-S., Lee J.-Y., Lee J.-S. (2015). The traditional drug Gongjin-Dan ameliorates chronic fatigue in a forced-stress mouse exercise model. *Journal of Ethnopharmacology*.

[15] Choi K. H., Park C. S. (2007). An analysis of the Gongjindan`s ingredients and its efficacy on anti-oxidation. *The Korean Journal of Herbology*.

[16] Sunwoo Y. Y., Park S. I., Chung Y. A. (2012). A pilot study for the neuroprotective effect of Gongjin-dan on transient middle cerebral artery occlusion-induced ischemic rat brain. *Evidence-Based Complementary and Alternative Medicine*.

[17] Hwang S. M., Chung D. K. (2004). The effects of KongJin-dan (KJD) on the Alzheimer's disease model induced by CT105. *Journal of Oriental Neuropsychiatry*.

[18] Kim Y. H., Bae M. J. (1989). The effect of Gongjindan on the lipid metabolism in rats fed high fat-diet. *The Journal of East-West Medicines*.

[19] Jung H. C., Jang H. J., Sung W. Y., Lee S. H., Son J. H., Han S. H. (2004). A study of Gongjin-dan in patients with mild dementia of Alzheimer type. *Journal of Oriental Neuropsychiatry*.

[20] Lee D. S., Kim D. W. (2004). A effect of Gongchen-dan to anemia. *Journal of Kyungwon University Korean Medicine Institution*.

[21] Son M. J., Im H.-J., Kim Y.-E., Ku B., Lee J.-H., Son C.-G. (2016). Evaluation of the anti-fatigue effects of a traditional herbal drug, Gongjin-dan, under insufficient sleep conditions: Study protocol for a randomised controlled trial. *Trials*.

[22] Chan A. W., Tetzlaff J. M., Altman D. G. (2013). SPIRIT 2013 statement: defining standard protocol items for clinical trials. *Annals of Internal Medicine*.

[23] Gagnier J. J., Boon H., Rochon P., Moher D., Barnes J., Bombardier C. (2006). Reporting randomized, controlled trials of herbal interventions: an elaborated CONSORT statement. *Annals of Internal Medicine*.

[24] Xue Z., Liu C.-Z., Shi G.-X. (2013). Efficacy and safety of acupuncture for chronic dizziness: Study protocol for a randomized controlled trial. *Trials*.

[25] Association of department of pathology in Korea medicine (1998). *Pathology in Korean Medicine*.

[26] Jacobson G. P., Newman C. W. (1990). The development of the Dizziness Handicap Inventory. *JAMA Otolaryngology–Head & Neck Surgery*.

[27] Han G. C., Lee E. J., Lee J. H. (2004). The study of standardization for a Korean adaptation of self-report measures of dizziness. *Journal of the Korean Balance Society*.

[28] Novotný M., Kostŕica R., Círek Z. (1999). The efficacy of Arlevert therapy for vertigo and tinnitus. *International Tinnitus Journal*.

[29] Novotný M., Kostřica R. (2002). Fixed combination of cinnarizine and dimenhydrinate versus betahistine dimesylate in the treatment of Meniere's disease: a randomized, double-blind, parallel group clinical study. *International Tinnitus Journal*.

[30] Scholtz A. W., Schwarz M., Baumann W., Kleinfeldt D., Scholtz H.-J. (2004). Treatment of vertigo due to acute unilateral vestibular loss with a fixed combination of cinnarizine and dimenhydrinate: A double-blind, randomized, parallel-group clinical study. *Clinical Therapeutics*.

[31] Cirek Z., Schwarz M., Baumann W., Novotny M. (2005). Efficacy and tolerability of a fixed combination of cinnarizine and dimenhydrinate versus betahistine in the treatment of otogenic vertigo: A double-blind, randomised clinical study. *Clinical Drug Investigation*.

[32] Pytel J., Nagy G., Tóth A., Spellenberg S., Schwarz M., Répassy G. (2007). Efficacy and tolerability of a fixed low-dose combination of cinnarizine and dimenhydrinate in the treatment of vertigo: A 4-week, randomized, double-blind, active- and placebo-controlled, parallel-group, outpatient study. *Clinical Therapeutics*.

[33] Hahn A., Sejna I., Stefflova B., Schwarz M., Baumann W. (2008). A fixed combination of cinnarizine/dimenhydrinate for the treatment of patients with acute vertigo due to vestibular disorders: A randomized, reference-controlled clinical study. *Clinical Drug Investigation*.

[34] Otto V., Fischer B., Schwarz M., Baumann W., Preibisch-Effenberger R. (2008). Treatment of vertebrobasilar insufficiency-associated vertigo with a fixed combination of cinnarizine and dimenhydrinate. *The International Tinnitus Journal*.

[35] Hahn A., Novotný M., Shotekov P. M., Cirek Z., Bognar-Steinberg I., Baumann W. (2011). Comparison of cinnarizinedimenhydrinate fixed combination with the respective monotherapies for vertigo of various origins: A randomized, double-blind, active-controlled, multicentre study. *Clinical Drug Investigation*.

[36] Toupet M., Ferrary E., Grayeli A. B. (2011). Visual analog scale to assess vertigo and dizziness after repositioning maneuvers for benign paroxysmal positional vertigo. *Journal of Vestibular Research*.

[37] Chiu C. W., Lee T. C., Hsu P. C. (2015). Efficacy and safety of acupuncture for dizziness and vertigo in emergency department: a pilot cohort study. *BMC Complementary and Alternative Medicine*.

[38] Reid S. A., Rivett D. A., Katekar M. G., Callister R. (2008). Sustained natural apophyseal glides (SNAGs) are an effective treatment for cervicogenic dizziness. *Manual Therapy*.

[39] Reid S. A., Rivett D. A., Katekar M. G., Callister R. (2012). Efficacy of manual therapy treatments for people with cervicogenic dizziness and pain: Protocol of a randomised controlled trial. *BMC Musculoskeletal Disorders*.

[40] Reid S. A., Rivett D. A., Katekar M. G., Callister R. (2014). Comparison of mulligan sustained natural apophyseal glides and maitland mobilizations for treatment of cervicogenic dizziness: A randomized controlled trial. *Physical Therapy in Sport*.

[41] Reid S. A., Callister R., Snodgrass S. J., Katekar M. G., Rivett D. A. (2015). Manual therapy for cervicogenic dizziness: Long-term outcomes of a randomised trial. *Manual Therapy*.

[42] Berg K., Wood-Dauphinee S., Williams J. I., Gayton D. (1989). Measuring balance in the elderly: preliminary development of an instrument. *Physiotherapy Canada*.

[43] Berg K., Wood-Dauphinee S., Williams J. I. (1995). The balance scale: reliability assessment with elderly residents and patients with an acute stroke. *Scandinavian Journal of Rehabilitation Medicine*.

[44] Berg K. O., Wood-Dauphinee S. L., Williams J. I., Maki B. (1992). Measuring balance in the elderly: validation of an instrument. *Canadian Journal of Public Health*.

[45] Thorbahn L. D. B., Newton R. A. (1996). Use of the Berg balance test to predict falls in elderly persons. *Physical Therapy in Sport*.

[46] Jung H. Y., Park J. H., Shim J. J., Kim M. J., Hwang M. R., Kim S. H. (2006). Reliability test of Korean version of berg balance scale. *Annals of Rehabilitation Medicine*.

[47] Hewlett S., Dures E., Almeida C. (2011). Measures of fatigue: Bristol rheumatoid arthritis fatigue multi-dimensional questionnaire (BRAF MDQ), Bristol rheumatoid arthritis fatigue numerical rating scales (BRAF NRS) for severity, effect, and coping, Chalder fatigue questionnaire (CFQ), checklist individual strength (CIS20R and CIS8R), fatigue severity scale (FSS), functional assessment chronic illness therapy (Fatigue) (FACIT-F), multi-dimensional assessment of fatigue (MAF), multi-dimensional fatigue inventory (MFI), pediatric quality of life (PedsQL) multi-dimensional fatigue scale, profile of fatigue (ProF), short form 36 vitality subscale (SF-36 VT), and visual analog scales (VAS). *Arthritis Care and Research*.

[48] Kamper S. J., Ostelo R. W. J. G., Knol D. L., Maher C. G., de Vet H. C. W., Hancock M. J. (2010). Global Perceived Effect scales provided reliable assessments of health transition in people with musculoskeletal disorders, but ratings are strongly influenced by current status. *Journal of Clinical Epidemiology*.

[49] Staab J. P., Ruckenstein M. J. (2005). Chronic dizziness and anxiety: Effect of course of illness on treatment outcome. *Archives of Otolaryngology—Head and Neck Surgery*.

[50] Rhee M. K., Lee Y. H., Jung H. Y. (1995). A standardization study of beck depression inventory II - Korean version (K-BDI): validity. *The Korean Journal of Psychopathology*.

[51] Rhee M. K., Lee Y. H., Park S. H. (1995). A standardization study of beck depression inventory I - Korean version (K-BDI): reliability and factor analysis. *The Korean Journal of Psychopathology*.

[52] Julian L. J. (2011). Measures of anxiety. *Arthritis Care and Research*.

[53] Woo H., Kim S., Lee S., Choi M., Kim Y., Lee J. (2008). Development of questionnaires for differentiation of qì-xū, xuè-xū, yang-xū, yīn-xū analysis. *The Journal of Internal Korean Medicine*.

[54] Kim J. H., Ku B. C., Kim J. E., Kim Y. S., Kim K. H. (2014). Study on reliability and validity of the “qi blood yin yang deficiency questionnaire”. *Korean Journal of Oriental Physiology & Pathology*.

[55] Kim J., Ku B., Kim K. H. (2016). Validation of the qi blood yin yang deficiency questionnaire on chronic fatigue. *Chinese Medicine*.

[56] Bang H., Ni L., Davis C. E. (2004). Assessment of blinding in clinical trials. *Controlled Clinical Trials*.

[57] Kim T. H. (2012). *Validity and reliability evaluation for EQ-5D in the general population of South Korea*.

[58] Wu X. (2011). *Clinical research on traditional acupuncture manipulation combined with the moxibustion on BaiHui of Meniere’s disease*.

